# Effects of Anthocyanin Supplementation on Serum Lipids, Glucose, Markers of Inflammation and Cognition in Adults With Increased Risk of Dementia – A Pilot Study

**DOI:** 10.3389/fgene.2019.00536

**Published:** 2019-06-11

**Authors:** Anne Katrine Bergland, Hogne Soennesyn, Ingvild Dalen, Ana Rodriguez-Mateos, Rolf Kristian Berge, Lasse Melvaer Giil, Lawrence Rajendran, Richard Siow, Michele Tassotti, Alf Inge Larsen, Dag Aarsland

**Affiliations:** ^1^Centre for Age-Related Medicine, Stavanger University Hospital, Stavanger, Norway; ^2^Department of Clinical Science, University of Bergen, Bergen, Norway; ^3^Section of Biostatistics, Department of Research, Stavanger University Hospital, Stavanger, Norway; ^4^Department of Nutritional Sciences, Faculty of Life Sciences and Medicine, School of Life Course Sciences, King’s College London, London, United Kingdom; ^5^The Lipid Research Group, Department of Clinical Science, University of Bergen, Bergen, Norway; ^6^Department of Internal Medicine, Haraldsplass Deaconess Hospital, Bergen, Norway; ^7^UK Dementia Research Institute, Institute of Psychiatry, Psychology & Neuroscience, King’s College London, London, United Kingdom; ^8^School of Cardiovascular Medicine and Sciences, British Heart Foundation Centre of Research Excellence, Faculty of Life Sciences and Medicine, King’s College London, London, United Kingdom; ^9^Department of Food & Drug, University of Parma, Parma, Italy; ^10^Department of Cardiology, Stavanger University Hospital, Stavanger, Norway

**Keywords:** mild cognitive impairment, MCI, anthocyanins, lipids, inflammation markers

## Abstract

**Background:**

Anthocyanins may protect against cardiovascular related cognitive decline and dementia.

**Objective:**

Open-label study to measure changes in serum lipids, glucose, glycosylated hemoglobin (HbA1c), and markers of inflammation after anthocyanin supplementation in people with increased risk of dementia. As a secondary endpoint we examined potential changes in a battery of cognitive test in the anthocyanin group (AG). A total of 27 individuals with mild cognitive impairment (MCI) (*n* = 8) or stable non-obstructive coronary artery disease (CAD) (*n* = 19) consumed two Medox^®^ capsules, each containing 80 mg of natural purified anthocyanins, twice daily for 16 weeks. They provided blood samples and performed a short battery of cognitive tests. Twenty healthy normal controls (NC) (*n* = 20) provided blood samples, but did not receive any intervention and did not perform cognitive tests.

**Results:**

There was a significant difference between groups for monocyte chemoattractant protein (MCP-1) and fasting glucose. In addition, total cholesterol and triglycerides were significantly increased in the AG. Improvements in memory and executive test scores were observed. No adverse effects were reported.

**Conclusion:**

The results of this pilot study were largely inconclusive with regard to the potential protective effects of anthocyanin supplementation. However, anthocyanins were well tolerated, and compliance was high. Larger, placebo-controlled studies to explore the potential effects of anthocyanins on dementia risk are encouraged.

**Clinical Trial Registration:**

www.ClinicalTrials.gov, identifier NCT02409446

## Introduction

Anthocyanins, a subclass of the flavonoids, are found in foods such as berries and fruits and information regarding their content in food can be found in an online phenol-explorer (Neveu et al., 2010). Anthocyanins have been shown in previous studies to have a number of positive health effects, such as improving the blood lipid profile (Qin et al., 2009; Li et al., 2015), and also fasting serum glucose and glycosylated hemoglobin (HbA1c) in diabetic patients (Li et al., 2015; Yang et al., 2017), and have anti-inflammatory effects (Xia et al., 2007; Spencer et al., 2012; Spagnuolo et al., 2017). Anthocyanins can improve endothelial and vascular function (Rodriguez-Mateos et al., 2013, 2016, 2019), and can cross the blood–brain barrier (Faria et al., 2014) and thus may reduce neurodegenerative and cerebrovascular changes and possibly protect against cognitive decline and dementia. Interestingly, some studies have found that food-based anthocyanins can improve memory functioning in older adults with mild cognitive impairment (MCI; Krikorian et al., 2010a,b; Hein et al., 2019). However, these studies were based on relatively small samples, and had a short duration. In addition, food-based anthocyanin supplementation leads to heterogeneity, i.e., variations in types of food sources, concentration, and dose of anthocyanins.

In this exploratory open-label pilot study, we aimed as a primary endpoint to examine potential changes in dementia-relevant mechanisms after 16 weeks of treatment with purified anthocyanin containing capsules, in people with increased risk of dementia. As a secondary endpoint we also explored the potential change in a battery of cognitive tests.

## Materials and Methods

### Material

Participants were recruited from the outpatient Memory and Cardiology clinics at Stavanger University Hospital in Norway during 2015 and 2016. Eligible for this study were patients with MCI or mild dementia and/or stable non-obstructive coronary artery disease (CAD). Potential participants identified at the respective outpatient clinics were contacted for a telephone interview by a study doctor, regarding inclusion and exclusion criteria. Participants were also recruited from the dementia disease initiation (DDI) study (Fladby et al., 2017). Inclusion criteria were age ≥50 years and being on stable medication, including nutraceuticals for the past 3 months, and either (a) confirmed CAD without physiologically significant stenosis evaluated by coronary angiography, or (b) having MCI or mild dementia according to ICD 10 (World Health Organization [WHO], 1992).

Exclusion criteria were moderate to severe dementia [operationalized as a mini-mental status exam (MMSE) (Folstein et al., 1975) score < 24], clinically significant depression [15–item Geriatric Depression Scale (GDS-15) (Mitchell et al., 2010) score ≥ 7], unstable CAD, heart failure in need of treatment, having taken Medox^®^ during the past 3 months, using Warfarin, heparin or non-vitamin K antagonist oral anticoagulants (NOAC), inflammatory illnesses such as rheumatoid arthritis and other severe illness with <5 years expected survival. Any treatment with vitamins, minerals or nutraceuticals had to have remained stable for the last 3 months prior to inclusion and during the study. Healthy normal controls (NC) (*n* = 20) recruited from the staff at Stavanger University Hospital and through acquaintances, were ≥50 years, had stable medication, and had not been taking Medox^®^ for the last 3 months. This group provided blood samples at inclusion and 16 weeks later, but did not take Medox^®^ or other interventions, and did not perform the cognitive test battery. The rationale for including this comparison group was to have a reference group with respect to any observed changes in the blood analyses in the intervention group.

### Ethics Statement

All participants provided written informed consent, and the study has been approved by the Regional Ethics Committee (Approval 2014/1966). The study has been registered at ClinicalTrials.gov (NCT02409446).

### Intervention, Design, and Assessment

#### Intervention

The participants were given open-label Medox^®^ capsules, provided free of charge by the manufacturer Medpalett AS, Sandnes, Norway. Medox^®^ capsules, which contain specific quantities of natural purified anthocyanins from bilberry (*Vaccinium myrtillus*) and blackcurrant (*Ribes nigrum*), have been used previously in human studies (Karlsen et al., 2007). The production of the Medox^®^ capsule (Hassellund et al., 2013), and its anthocyanin content (Qin et al., 2009) have been described previously.

The capsules were dispensed at inclusion in the study, and the participants were instructed to consume two 80 mg anthocyanin capsules twice daily for a total daily intake of 320 mg anthocyanins for 16 weeks. This dosage was chosen because it has previously been shown to have biological effects (Qin et al., 2009; Zhu et al., 2011; Li et al., 2015) and found to be safe in use (Qin et al., 2009). A review found that doses up to 640 mg/day showed no adverse events (Wallace et al., 2016).

Participants were instructed to maintain their dietary and lifestyle habits in order to avoid interferences in the study results.

#### Design and Assessment

At inclusion, all participants underwent a physical examination, including standardized blood pressure measurement, electrocardiogram (ECG), and blood tests. In addition a cognitive test battery (see below) was administered, including the MMSE and GDS-15. Following standardized procedures, participants provided blood samples in the morning after having been fasting for at least 8 h, before and after 16 weeks of treatment. They were contacted by telephone after 8 weeks regarding safety and compliance.

### Blood Sampling and Analyses

Blood was collected, centrifugated, and stored at −80 °C until analysis according to standardized procedures. The serum samples were analyzed for lipids (total cholesterol, triglycerides, HDL- and LDL cholesterol) and fasting glucose using Architect c16000 TM (Abbott Diagnostics, Chicago, IL, United States) and HbA1c using Variant II turbo (BioRad, Hercules, CA, United States) at Stavanger University Hospital.

Markers of inflammation were analyzed after completion of the study by The Lipid Research Group, Department of Clinical Sciences, University of Bergen, Bergen, Norway. Concentrations of cytokines were measured in serum using the Bio-Plex Pro^TM^ Human Cytokine 8-plex assay (Cat.: M50000007A) which included GM-CSF, IFN-γ, IL-2, IL-4, IL-6, IL-8, IL-10, and TNF-α, in addition to five Bio-Plex Pro Human Cytokine single-plexes: MCP-1 (Cat.: 171B5021M), RANTES (Cat.: 171B5025M), G-CSF (Cat.: 171B5017M), IL-17 (Cat.: 171B5014M), and IL-Iβ (Cat.:171B5001M). All plexes were manufactured by Bio-Rad (Hercules, CA, United States). The cytokines were detected by the Bio-Plex^TM^ 200 System and determined with the Bio-Plex Manager Software 6.1. The samples were prepared as described in the protocol (Cat.: 10014905) with a dilution factor of three.

Anthocyanin metabolites were measured in plasma after completion of the study at Department of Nutritional Sciences, School of Life Course Sciences, Faculty of Medicine and Life Sciences, King’s College London, using a method based on microelution solid phase extraction followed by liquid chromatography and mass spectrometry, using authentic standards, as previously described with some modifications (Feliciano et al., 2016). The detection of plasma (poly)phenol metabolites was performed on a Exactive^TM^ Orbitrap Mass Spectrometer (Thermo Scientific, Waltham, CA, United States) after separation on an Accela 1250 pump UHPLC system (Thermo Scientific, Waltham, CA, United States). The autosampler injected 5 μL of each sample in a Zorbax Eclipse Plus RRHD column 2.1 mm × 50 mm, 1.8 m with a compatible Eclipse Plus guardcolumn 2.1 mm × 5 mm, 1.8 m (Agilent, Waldbronn, Germany). The mobile phase consisted of 0.1% HCOOH (solvent A) and acetonitrile with 0.1% HCOOH (solvent B) in a 10 min gradient program. Quantification analysis of the plasma (poly)phenols was done using Xcalibur 2.2 (Thermo Scientific, Waltham, CA, United States).

### Cognitive Tests

Verbal memory function was assessed using the Norwegian adaptation of the Ten Word List Learning and Recall from the CERAD battery (Morris et al., 1989), a three part test; word list learning, word list delayed recall, and word list delayed recognition.

Executive functioning was assessed by the Trail Making Test (TMT) A and B (Reitan and Wolfson, 1985) and Stroop Golden (Golden, 1976). The Trail Making Tests A and B are tests of psychomotor speed and attention shifting (Ashendorf et al., 2008), while Stroop Golden is a test used to evaluate cognitive speed and inhibition (Scarpina and Tagini, 2017).

### Safety

Participants were contacted by phone at week 8 to ask about potential side-effects and adverse events (AE).

The safety blood tests included hemoglobin, thrombocytes, kidney function and liver function tests, which were measured at both baseline and study-end.

### Compliance

Participants were contacted by phone at week 8 and asked about adherence to the protocol. Specifically, they were asked whether they had been taking Medox^®^ capsules as instructed, and they were reminded about keeping the empty blister packages. Protocol adherence was also assessed by collecting and counting the empty blister packages and left-over capsules at study-end.

### Statistical Analyses

Descriptive statistics are presented as medians and interquartile ranges (IQR), and illustrated using Box plots. Most data were not normally distributed and thus the main analyses were non-parametric. The Mann–Whitney *U* test and the Chi-square test were used for between-group comparisons. Changes from baseline to follow-up at 24 weeks within groups were analyzed with the Wilcoxon Signed Rank test. For all tests *p* ≤ 0.05 was considered statistically significant.

Supplementary parametric analyses were performed, from which we present means, standard deviations (SD), and *p*-values from paired and independent samples *t*-tests.

The IBM SPSS statistical package version 24 was used for all statistical analyses.

## Results

**FIGURE 1:**
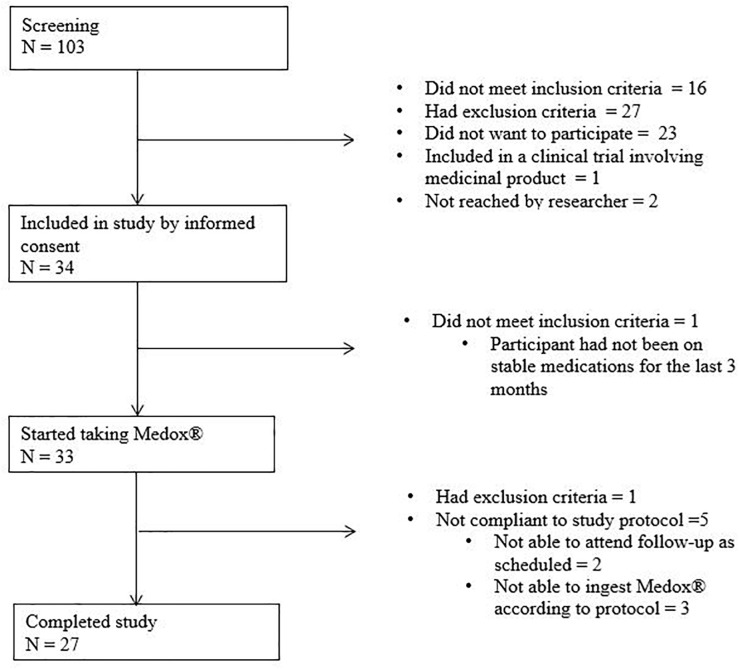
Enrolment of participants. N, number.

During the period May 2015 to September 2016, 33 participants started anthocyanin supplementation, of whom 27 (8 MCI and 19 CAD) completed the study (6 were excluded for administrative and logistical reasons) (Figure 1). The NC were included in the period December 2016 to February 2017. No subjects with mild dementia were included.

Baseline characteristics are shown in Table 1.

**Table 1:** Baseline characteristics.

	Active (*n* = 27)	Controls (*n* = 20)
	Median (IQR)	Median (IQR)
Women, count (%)	9 (33)	11 (55)
Age (years)	61 (55–70)	58 (55–62)
CAD, count (%)	19 (70)	
Education (years)	11.5 (10–14)	
BMI	27.7 (26.0–30.3)	
Lipid lowering therapy, count (%)	19 (70)	
Acetylsalicylic acid, count (%)	17 (63)	
Oral antidiabetic treatment count (%)	3 (11)	
Dietary supplement, count (%)	18 (67)	

*CAD, coronary artery disease; BMI, body mass index; IQR, interquartile range.*

Only IL-8, MCP-1, CCL-5/RANTES [regulated on activation, normal T-cell expressed and secreted (RANTES)] and TNF were available for statistical analyses, as the other inflammation markers did not reach measurement thresholds. The findings are summarized in Table 2 and Supplementary Figures S1–S3.

**TABLE 2:** Changes from baseline to 16 weeks follow-up in serum variables, for participants with supplementation (active) and for control participants.

	**Active (*n* = 27)**		**Control (*n* = 20)**		**Active vs. Control**
					
	**Median (IQR)**	p*	**Median (IQR)**	p*	***p*^#^**
**Cholesterol (mmol/L)**			**Cholesterol**		
Pre	4.0 (3.1 to 5.5)		5.1 (4.5 to 5.5)		
Post	4.6 (3.3 to 6.0)		5.1 (4.6 to 5.6)		
Diff	0.2 (0.1 to 0.7)	0.009	0.1 (−0.2 to 0.5)	0.29	0.34
**HDL (mmol/L)**			**HDL**		
Pre	1.2 (1.0 to 1.4)		1.5 (1.1 to 1.7)		
Post	1.2 (1.1 to 1.5)		1.4 (1.2 to 1.8)		
Diff	0.0 (−0.1 to 0.1)	0.81	0.1 (−0.1 to 0.1)	0.21	0.23
**LDL (mmol/L)**			**LDL^*n* = 19^**		
Pre	2.4 (1.8 to 3.9)		3.3 (2.9 to 3.9)		
Post	3.0 (1.8 to 4.3)		3.3 (2.8 to 4.0)		
Diff	0.1 (−0.1 to 0.3)	0.21	0.0 (−0.1 to 0.4)	0.62	0.72
**Triglycerides (mmol/L)**			**Triglycerides**		
Pre	1.0 (0.7 to 1.4)		0.9 (0.6 to 1.3)		
Post	1.0 (0.7 to 1.7)		0.9 (0.6 to 1.8)		
Diff	0.1 (0.7 to 1.7)	0.016	0.0 (−0.1 to 0.4)	0.072	0.84
**Fasting glucose (mmol/L)**			**Fasting glucose**		
Pre	5.4 (4.9 to 5.6)		5.3 (5.0 to 5.6)		
Post	5.5 (5.3 to 6.3)		5.0 (4.8 to 5.7)		
Diff	0.2 (−0.1 to 0.4)	0.058	−0.2 (−0.4 to −0.03)	0.009	***0.003***
**HbA1c (%)**			**HbA1c**		
Pre	5.8 (5.6 to 6.1)		5.6 (5.4 to 5.8)		
Post	5.8 (5.6 to 6.1)		5.4 (5.2 to 5.6)		
Diff	0.0 (−0.1 to 0.1)	0.87	−0.05 (−0.2 to 0.0)	0.057	***0.26***
**IL-8 (mmol/L)**			**IL−8**		
Pre	9.0 (7.7 to 10.3)		7.5 (7.2 to 8.4)		
Post	9.2 (6.9 to 11.1)		7.8 (7.2 to 8.9)		
Diff	0.0 (−1.5 to 1.2)	0.80	0.2 (−1.0 to 1.5)	0.79	***0.71***
**MCP-1 (pg/mL)**			**MCP-1**		
Pre	42.2 (10.3 to 59.4)		51.7 (40.9 to 70.2)		
Post	41.3 (11.1 to 60.2)		52.8 (45.1 to 93.7)		
Diff	0.0 (−5.4 to 1.7)	0.55	1.9 (0.2 to 17.5)	0.014	***0.011***
**RANTES (pg/mL)**			**RANTES**		
Pre	9206 (8172 to 9833)		8800 (8370 to 9761)		
Post	8918 (8046 to 9942)		9164 (8651 to 10027)		
Diff	−161 (−730 to 677)	0.81	19.09 (−633 to 1105)	0.41	***0.41***
**TNFa (pg/mL)**			**TNFa^*n* = 19^**		
Pre	10.1 (7.8 to 13.3)		6.5 (6.1 to 10.9)		
Post	9.9 (6.5 to 13.9)		8.0 (5.8 to 11.4)		
Diff	0.9 (−2.8 to 2.9)	0.74	−0.4 (−1.5 to 3.9)	0.66	***0.95***

*RANTES; CCL-5/RANTES (regulated on activation, normal T-cell expressed and secreted); Diff, median difference between baseline and follow up serum measurements; IQR, interquartile range; mmol/L, millimol/liter; pg/mL, picomol/liter.* *The within group difference from baseline to study end.^#^*The between group differences for* Δ *(difference from baseline to study end).*

The only significant between-group difference was for difference were for ΔMCP-1 (difference from baseline to study end) (*p* = 0.011) and Δfasting glucose (*p* = 0.003) (Table 2).

When analyzing the groups separately, significant increases were found in total cholesterol and triglycerides in the anthocyanin supplementation group (AG) from baseline to study end (Table 2), and MCP-1 which increased in the NC group (Table 2).

No significant changes were found for fasting glucose and HbA1c in the AG group.

A total of 29 plasma anthocyanin metabolites were quantified (Table 3 and Supplementary Figure S4). When comparing the two groups, a statistically significant difference was found for two metabolites (*o*-Coumaric acid and Dihydroferulic acid-4-*O*-Sulfate), which both had a larger decrease in the AG than in the NC group (Table 3).

**Table 3:** Changes from baseline to 16 weeks follow-up in plasma anthocyanin metabolites, for participants with supplementation (active) and for control participants.

	Active (*n* = 27)		Control (*n* = 20)		Active vs. control
nmol/L	Median (IQR)	*p^∗^*	Median (IQR)	*p^∗^*	*p^#^*
**Methylpyrogallol-*O*-sulfate**					
Pre	33.1 (17.5 to 52.5)		26.2 (10.2 to 67.6)		
Post	45.5 (24.1 to 104.2)		20.6 (7 to 65.3)		
Diff	15.6 (−3.3 to 70.7)	0.068	−1.7 (−23.8 to 13.0)	0.85	0.14
**Pyrogallol-2-*O*-sulfate**					
Pre	36.4 (17.2 to 110.5)		58.4 (20.4 to 102.4)		
Post	63.8 (41.5 to 199.1)		35.2 (21.2 to 93.1)		
Diff	17.5 (−8.3 to 106.4)	0.001	−12.3 (−82.4 to 32.1)	0.41	0.89
**Protocatechuic acid-3-*O*-sulfate**					
Pre	3.5 (0.3 to 11.1)		5.4 (1.6 to 10.1)		
Post	11.0 (5.5 to 26.5)		6.5 (0.3 to 16.4)		
Diff	6.2 (0.0 to 21.6)	0.007	0.8 (−6.3 to 10.1)	0.81	0.071
**1-Methylpyrogallol-*O*-sulfate**					
Pre	44.7 (29.2 to 77.3)		60.8 (34.8 to 123)		
Post	52.7 (33.6 to 97.2)		71.8 (30.3 to 106)		
Diff	12.8 (−19.6 to 32.7)	0.14	10.4 (−27.8 to 65.5)	0.60	0.70
**4-Methylgallic-3-*O*-sulfate**					
Pre	14.0 (5.6 to 23.8)		13.1 (8.5 to 28.7)		
Post	12.8 (7.1 to 24.6)		12.3 (7.3 to 21.1)		
Diff	1.6 (−7.8 to 14.3)	0.65	−5.5 (−15.6 to 12.0)	0.35	0.25
**4-Hydroxybenzoic acid-*O*-sulfate**					
Pre	2027 (1211 to 4284)		2664 (1455 to 3764)		
Post	1704 (772 to 3597)		1723 (420 to 4354)		
Diff	−97 (−1039 to 234)	0.14	−924 (−2691 to 1046)	0.26	0.67
**4-Hydroxyhippuric acid**					
Pre	164 (93 to 272)		145 (117 to 275)		
Post	123 (94 to 283)		111 (91 to 163)		
Diff	6 (−84 to 44)	0.84	−33 (−236 to 33)	0.079	0.21
**Protocatechuic acid**					
Pre	57.5 (22.0 to 106.8)		66.6 (35.5 to 127)		
Post	51.3 (29.4 to 114.2)		48.7 (5.0 to 76.9)		
Diff	1.6 (−49.7 to 51.3)	0.75	−22.0 (−85.2 to 17.2)	0.049	0.057
**Pyrogallol-1-*O*-sulfate**					
Pre	25.6 (15.3 to 61.2)		55.1 (25.3 to 106.5)		
Post	52.9 (25.5 to 97.6)		63.8 (41.4 to 94.4)		
Diff	18.8 (1.5 to 68.4)	0.006	−2.9 (−36.3 to 45.7)	0.85	0.093
**3,4-Dihydroxyphenylacetic acid**					
Pre	174 (100 to 237)		122 (85 to 197)		
Post	106 (84 to 213)		98 (75 to 163)		
Diff	−26 (−135 to 69)	0.20	−33 (−67 to 2)	0.044	0.97
**Catechol-*O*-sulfate**					
Pre	5518 (2718 to 8056)		5986 (4284 to 7527)		
Post	4640 (3183 to 7843)		4690 (3576 to 5627)		
Diff	367 (−2569 to 1608)	0.61	−937 (−3502 to 2010)	0.25	0.67
**Vanillic acid-4-*O*-sulfate**					
Pre	30.6 (9.5 to 47.8)		24.6 (9.6 to 39.6)		
Post	26.2 (9.8 to 42.3)		22.6 (7.9 to 34.7)		
Diff	−6.5 (−27.6 to 10.8)	0.20	−5.2 (−15.0 to 23.8)	1.0	0.41
**3-Hydroxyhippuric acid**					
Pre	709 (191 to 3060)		451 (269 to 1343)		
Post	339 (63 to 1233)		162 (35 to 599)		
Diff	−503 (−1246 to −55)	0.002	−183 (−1117 to 196)	0.10	0.28
***p*-Coumaric acid-4-*O*-β-*D*-glucuronide**					
Pre	0.29 (0.00 to 0.52)		0.23 (0.00 to 0.46)		
Post	0.21 (0.11 to 0.41)		0.17 (0.00 to 0.30)		
Diff	−0.07 (−0.26 to 0.24)	0.92	0.01 (−0.20 to 0.20)	0.94	0.84
**Isovanillic acid-3-*O*-sulfate**					
Pre	2.3 (0.0 to 8.3)		5.5 (0.0 to 19.1)		
Post	2.8 (0.0 to 11.3)		5.7 (0.3 to 14.4)		
Diff	0.0 (−3.5 to 11.3)	0.90	−1.1 (−10.6 to 3.6)	0.50	0.48
**Catechol-*O*-1-glucuronide**					
Pre	1.7 (0.0 to 9.2)		2.2 (0.1 to 8.0?)		
Post	4.6 (1.6 to 14.2)		1.1 (0.1 to 6.6)		
Diff	1.5 (−1.5 to 8.8)	0.075	0.1 (−2.2 to 1.7)	0.97	0.14
**Ferulic acid-4-*O*-β-*D*-glucuronide**					
Pre	4.3 (0.4 to 33.4)		7.7 (1.0 to 19.4)		
Post	19.4 (2.6 to 59.3)		5.8 (1.1 to 21.5)		
Diff	5.5 (0.2 to 31.4)	0.013	−0.3 (−5.9 to 15.4)	0.98	0.064
**Hippuric acid**					
Pre	17680 (9444 to 55008)		24075 (11958 to 39646)		
Post	15225 (8654 to 40673)		12211 (11086 to 28936)		
Diff	−3572 (−18398 to 1041)	0.068	−2788 (−15907 to 4975)	0.33	0.68
**4-Methylcatechol-*O*-sulfate**					
Pre	1630 (815 to 3580)		1209 (753 to 1889)		
Post	1228 (691 to 2722)		997 (647 to 1899)		
Diff	−182 (−1087 to 733)	0.47	−221 (−617 to 255)	0.28	0.95
**4-Hydroxybenzaldehyde**					
Pre	72.6 (40.3 to 130)		81.2 (58.4 to 105)		
Post	53.5 (48.9 to 93.3)		48.7 (39.5 to 75.5)		
Diff	−24.8 (−56.1 to 6.5)	0.029	−34.3 (−52.8 to −6.8)	0.019	0.78
**Ferulic acid-4-*O*-sulfate**					
Pre	2.3 (0.5 to 7.0)		4.0 (0.0 to 12.3)		
Post	1.9 (0.8 to 23.8)		6.1 (1.6 to 9.7)		
Diff	0.5 (−2.9 to 21.1)	0.20	1.1 (−7.7 to 9.1)	0.55	0.69
**Dihydroisoferulic acid-3-*O*-sulfate**					
Pre	7.2 (0.0 to 17.5)		4.9 (0.0 to 14.1)		
Post	9.2 (2.1 to 23.7)		7.6 (1.0 to 19.4)		
Diff	1.0 (−7.5 to 8.6)	0.47	0.9 (−6.9 to 13.0)	0.69	0.94
**Isoferulic acid-3-*O*-sulfate**					
Pre	1.1 (0.3 to 2.3)		0.8 (0.4 to 5.7)		
Post	4.5 (0.4 to 13.7)		1.5 (0.3 to 8.6)		
Diff	0.6 (−1.7 to 10.2)	0.25	0.4 (−1.6 to 2.4)	0.55	0.67
**Dihydroisoferulic acid-3-*O*-β-*D*-glucuronide**					
Pre	19.1 (7.2 to 45.7)		25.8 (2.5 to 82.4)		
Post	20.7 (8.1 to 60.8)		16.1 (5.4 to 43.4)		
Diff	1.2 (−15.4 to 34.5)	0.47	−4.9 (−70.9 to 27.7)	0.55	0.31
**Isoferulic acid-3-*O*-β-*D*-glucuronide**					
Pre	22.3 (5.2 to 67.5)		21.2 (1.8 to 54.6)		
Post	27.3 (8.5 to 136.4)		21.3 (5.7 to 58.6)		
Diff	24.4 (−18.0 to 96.2)	0.044	2.7 (−10.1 to 36.8)	0.55	0.21
**Dihydroferulic acid-4-*O*-sulfate**					
Pre	3.7 (1.2 to 8.3)		1.9 (0.2 to 3.6)		
Post	1.6 (0.9 to 5.1)		2.7 (1.4 to 5.2)		
Diff	−1.5 (−3.1 to −0.3)	0.006	0.8 (−0.9 to 2.9)	0.31	0.010
**3-(3-hydroxyphenyl)propanoic acid**					
Pre	805 (44 to 2377)		350 (14 to 1479)		
Post	470 (72 to 4306)		414 (112 to 1624)		
Diff	29 (−495 to 831)	0.43	36 (−917 to 345)	0.74	0.76
***m*-Coumaric acid**					
Pre	111 (35 to 146)		84 (63 to 110)		
Post	55 (38 to 78)		58 (53 to 85)		
Diff	−62 (−82 to 16)	0.014	−28 (−58 to 2)	0.093	0.33
***o*-Coumaric acid**					
Pre	218 (81 to 356)		125 (81 to 234)		
Post	87 (47 to 158)		81 (50 to 162)		
Diff	−116 (−230 to −35)	<0.001	−36 (−111 to −1)	0.006	0.019

*Diff, median difference between baseline and follow up plasma measurements; IQR, interquartile range; nmol/L = nanomol/liter. ^∗^The within group difference from baseline to study end. ^#^The between group differences for Δ (difference from baseline to study end).*

In the AG, there was a statistically significant increase in five of the metabolites (Pyrogallol-2-*O*-sulfate, Protocatechuic acid-3-*O*-sulfate, Pyrogallol-1-*O*-sulfate, Ferulic acid-4-*O*-β-*D*-glucuronide, Isoferulic_acid-3-*O*-β-*D*-glucuronide) and a statistically significant decrease in five other metabolites (3-Hydroxyhippuric acid, 4-Hydroxybenzaldehyde, Dihydroferulic acid-4-*O*-Sulfate, *m*-Coumaric acid, *o*-Coumaric acid) after 16 weeks of anthocyanin consumption in comparison with baseline.

In the NC group, there was a statistically significant decrease in four metabolites (Protocatechuic acid, 3,4-Dihydroxyphenylacetic acid, 4-Hydroxybenzaldehyde and *o*-Coumaric acid), whereas there were no statistically significant increases in any of the metabolites.

The cognitive test scores improved in the intervention group, with improvements for CERAD learning (*p* = 0.016), recall (*p* < 0.001) and recognition (*p* = 0.047) and for STROOP test word (*p* < 0.001) and color (*p* = 0.044) (Table 4 and Supplementary Figures S5–S7).

**Table 4:** Changes from baseline to 16 weeks follow-up in cognitive variables, for participants with supplementation (active).

	Active (*n* = 27)	
	Median (IQR)	*p*^∗^
**CERAD (points) Learning**		
Pre	20 (16 to 22)	
Post	21 (17 to 25)	
Diff	2 (−1 to 3)	0.016
**Recall**		
Pre	6 (4 to 8)	
Post	7 (5 to 9)	
Diff	1 (0 to 2)	<0.001
**Recognition**		
Pre	20 (16 to 20)	
Post	20 (19 to 20)	
Diff	0 (0 to 1)	0.047
**TMT A (sec)**		
Pre	32 (21 to 53)	
Post	34 (23 to 39)	
Diff	−2 (−6 to 2)	0.081
**TMT B*^n^*^=19^ (sec)**		
Pre	85.50 (62.25 to 118)	
Post	69.50 (56 to 100.75)	
Diff	−2.0 (−19.75 to 2.25)	0.16
**STROOP (score) Word**		
Pre	87 (72 to 67)	
Post	87 (80 to 103)	
Diff	6 (1 to 10)	<0.001
**Color**		
Pre	61 (52 to 67)	
Post	62 (54 to 69)	
Diff	2 (−1 to 6)	0.044
**Word-color**		
Pre	34 (29 to 41)	
Post	34 (28 to 41)	
Diff	0 (−3 to 5)	0.67

*TMT, Trail Making Test; Diff, median difference between baseline and follow up results in cognitive tests; IQR, interquartile range. ^∗^The within group difference from baseline to study end.*

Overall, findings using parametric analyses differed only marginally from the non-parametric findings reported above (Supplementary Tables S1, S2).

The compliance was good. More than 85% of the participants returned at least 90% of the empty blister packages. The anthocyanins were well tolerated, and none of the participants withdrew due to adverse effects. Blood tests taken for safety reasons were all within a clinically acceptable range. Increased bleeding tendency was not observed.

## Discussion

In this pilot study anthocyanin supplementation was well tolerated, without any AE, and the compliance was good. This indicates that larger RCTs might be feasible, to confirm exploratory results in the current pilot study.

Our findings are somewhat inconclusive. While some cognitive improvements were observed in the AG, there were no significant changes in serum levels of some risk factors for dementia; i.e., fasting glucose, HbA1c or pro-inflammatory cytokines. There was a non-significant increase in serum levels of MCP-1 in the AG and a significant increase in the NC during the study period. The between-group difference in Δ serum levels of MCP-1 was statistically significant.

Furthermore, we observed a significant increase in serum levels of total cholesterol and triglycerides in AG. The lipid profile of the NC group did not change significantly, and since we have no information about statin use or use of other lipid lowering medications in the NC group, the observed difference should be interpreted cautiously.

Previous studies using Medox^®^ have shown a statistically significant increase in HDL-cholesterol (Qin et al., 2009; Zhu et al., 2011; Hassellund et al., 2013) and a decrease in LDL-cholesterol (Qin et al., 2009; Zhu et al., 2011). This is of clinical interest, as higher HDL-cholesterol is associated with lower cardiovascular risk (Barter et al., 2007), while high levels of total cholesterol, triglycerides and LDL are associated with higher cardiovascular risk (Stone et al., 2014).

The differences in the findings between our study and these previous studies might be due to differences in participants, as well as in anthocyanin supplementation dose and duration, or other factors. Furthermore, other studies included dyslipidemic and hypercholesterolemic participants not using statins or any other lipid lowering treatment (Qin et al., 2009; Zhu et al., 2011), whereas in our study, the median cholesterol at baseline in the intervention group was 4.0 mmol/l, in addition 70% were taking statins or other lipid lowering medication.

Regarding the inflammation markers, RANTES promotes activation and migration of leukocytes and mediates neuroinflammation and brain microvascular dysfunction (Appay and Rowland-Jones, 2001; Dénes et al., 2010; Yilmaz and Granger, 2010). As there was a significant between-group difference for ΔMCP-1, our results are partly consistent with findings in a randomized, double-blind trial in hypercholesterolemic individuals consuming purified anthocyanins for 24 weeks (Song et al., 2014), and in a parallel-designed, placebo-controlled trial (Karlsen et al., 2007). Other studies did not report a reduction of pro-inflammatory mediators after anthocyanin supplementation (Hassellund et al., 2013; Kent et al., 2015). Therefore, the anti-inflammatory effect of anthocyanins and the potential to reduce neuroinflammation and brain microvascular dysfunction associated with cognitive decline in adults at risk of dementia (Grammas, 2011) should be studied in larger randomized studies.

The beneficial effect of anthocyanins might possibly be due to their degradation products and metabolites (Feliciano et al., 2016) as absorption of intact anthocyanins is reported to be low (Zhong et al., 2017). Rodriguez-Mateos et al. quantified metabolites in plasma after blueberry consumption, and showed that anthocyanin-derived metabolites correlated with *in vivo* effects (Rodriguez-Mateos et al., 2013, 2019). Furthermore, circulating anthocyanin metabolites were shown to improve vascular function when injected in an *in vivo* model of vascular function, and cardiovascular benefits after consumption of anthocyanins were linked with anthocyanin metabolites as mediators of change in cellular gene programs (Rodriguez-Mateos et al., 2019).

In our study, we were able to measure a total of 29 anthocyanin metabolites. The results were conflicting, as we found various anthocyanin metabolites to be both significantly increasing and decreasing in the AG. In the NC group as well, we found significant changes. This is in line with findings in another study reporting an increase of some metabolites, and a decrease in others after ingestion of anthocyanins over time (Feliciano et al., 2016). A possible explanation for our results could be high inter-individual variability and the influence of the background diet on the concentration of these compounds in blood, as some of these metabolites could arise from the consumption of other anthocyanin-rich foods such as berries and red wine or other food components in the diet. It is also possible that the results could be related to the handling and analysis of the blood samples. As far as we know, the presence of anthocyanin metabolites in plasma after Medox^®^ use is reported in only one other clinical study, where 8 out of 17 anthocyanin metabolites were found to be significantly increased. However, this was analyzed in blood samples collected 1–3 h after ingestion of the morning dose of anthocyanins (320 mg), and not after daily consumption and an overnight fast (Hassellund et al., 2013).

The improvement on several cognitive tests should be interpreted cautiously due to potential learning effects related to the relatively short test–retest interval, and the lack of a comparison group. Nonetheless, our results are in line with previous smaller studies involving participants with MCI, reporting improved cognition after ingestion of anthocyanins (Krikorian et al., 2010a,b). Our results are also in line with the results of a randomized, double-blinded, placebo-controlled study reporting improved episodic memory after 3 months of anthocyanin supplementation (Whyte et al., 2018).

Intake of anthocyanin capsules in the dosage of 320 mg/day appears to be well tolerated and safe. None of the safety blood tests were found to be out of a clinically acceptable range or necessitating medical follow-up after study-end. This is consistent with previous studies (Karlsen et al., 2007; Qin et al., 2009).

The major limitations of this pilot study are the non-randomized open-label design, the small sample size, and the relatively short intervention period. Although all the participants were told not to change their lifestyle during the intervention period, we have no data on this. Further we had no detailed dietary assessment, and thus we cannot exclude the possibility of differences in the background diet between the groups before or during the study. However, the participants were instructed to maintain their dietary and lifestyle habits during the study period, and to take the capsules 30 min before or 120 min after meals, as concomitant ingestion of certain types of food may counteract the effect of flavonoids (Lorenz et al., 2007).

The NC were recruited separately and differed from the participants by being a healthy group that did not receive any intervention, and did not perform cognitive testing. Thus power calculations for further studies might be compromised. However, we provide descriptive statistics including variance measurements which may be helpful in planning sample size in later studies.

An important strength of our study is the well characterized combination of nutraceuticals used, which facilitates comparison with other studies regarding both source of anthocyanins and dosage. Of note, this is, to our knowledge, the first study on cognitive function in adults with increased risk of dementia where Medox^®^ is being used as the source of anthocyanin. Thus our study might also facilitate investigation of the effect of different proprietary blueberry formulatons, shown by Whyte et al. to be of importance (Whyte et al., 2018).

All things considered, adequately powered, randomized studies are warranted to better understand how anthocyanins and their metabolites may affect relevant mechanisms, including their possible protective role in epigenetic modifications that potentially benefit the aging brain and reduce the risk for dementia.

## Ethics Statement

All participants provided written informed consent, and the study has been approved by the Regional Ethics Committee (Approval 2014/1966). The study has been registered at ClinicalTrials.gov (NCT02409446).

## Author Contributions

DA, HS, AB, and AL planned and designed the study. AB conducted the study and collected the data. RB performed the serum analyses. MT and AR-M performed the plasma analyses. AB and ID conducted the statistical analyses. All authors wrote the manuscript and critically reviewed the manuscript.

## Conflict of Interest Statement

AB has received support for conference participation from Evonik. DA has received research support and/or honoraria from Astra-Zeneca, H. Lundbeck, Novartis Pharmaceuticals, and GE Health, and serves as paid consultant for H. Lundbeck and Axovant. The remaining authors declare that the research was conducted in the absence of any commercial or financial relationships that could be construed as a potential conflict of interest.

## Supplementary Material

FIGURE S1 Changes from baseline to 16 weeks follow-up in serum lipids, for participants with anthocyanin supplementation. mmol/L, millimole/liter.

FIGURE S2 Changes from baseline to 16 weeks follow-up in serum fasting glucose and HbA1c, for participants with anthocyanin supplementation. HbA1a, glycosylated hemoglobin; mmol/L, millimole/liter.

FIGURE S3 Changes from baseline to 16 weeks follow-up in serum cytokines, for participants with anthocyanin supplementation. RANTES;CCL-5/RANTES (regulated on activation, normal T-cell expressed and secreted). pg/mL, picomolar/milliliter.

FIGURE S4 Changes from baseline to 16 weeks follow-up in plasma anthocyanin metabolites, for participants with and without anthocyanin supplementation. Measurement unit: nmol/L, nanomolar/liter, except for hippuric acid (nmol/10L) and coumaric acid-4-*O*-β-*D*-glucuronide (nmol/0.033L).

FIGURE S5 Changes from baseline to 16 weeks follow-up in CERAD.

FIGURE S6 Changes from baseline to 16 weeks follow-up in Trail Making Test (TMT). Sec, seconds.

FIGURE S7 Changes from baseline to 16 weeks follow-up in Stroop test.

TABLE S1 Changes from baseline to 16 weeks follow-up in serum variables, for participants with supplementation (active) and for control participants. , mean; SD, standard deviation; ^∗^The within group difference from baseline to study end tested with paired-samples *t*-test. ^$^independent samples *t*-test for the between group differences for Δ (difference from baseline to study end). ^#^With Welch correction for heteroscedasticity. RANTES;CCL-5/RANTES (regulated on activation, normal T-cell expressed and secreted). Diff, mean difference between baseline and follow up results in serum measurements.

TABLE S2 Changes from baseline to 16 weeks follow-up in cognitive variables, for participants with supplementation (active). , mean; SD, standard deviation; TMT, Trail Making Test; Diff, mean difference between baseline and follow up results in cognitive tests. ^∗^The within group difference from baseline to study end tested with paired-samples *t*-test.
