# Corrigendum: Effects of Anthocyanin Supplementation on Serum Lipids, Glucose, Markers of Inflammation and Cognition in Adults With Increased Risk of Dementia – A Pilot Study

**DOI:** 10.3389/fgene.2021.678504

**Published:** 2021-05-31

**Authors:** Anne Katrine Bergland, Hogne Soennesyn, Ingvild Dalen, Ana Rodriguez-Mateos, Rolf Kristian Berge, Lasse Melvaer Giil, Lawrence Rajendran, Richard Siow, Michele Tassotti, Alf Inge Larsen, Dag Aarsland

**Affiliations:** ^1^Centre for Age-Related Medicine, Stavanger University Hospital, Stavanger, Norway; ^2^Department of Clinical Science, University of Bergen, Bergen, Norway; ^3^Section of Biostatistics, Department of Research, Stavanger University Hospital, Stavanger, Norway; ^4^Department of Nutritional Sciences, Faculty of Life Sciences and Medicine, School of Life Course Sciences, King's College London, London, United Kingdom; ^5^The Lipid Research Group, Department of Clinical Science, University of Bergen, Bergen, Norway; ^6^Department of Internal Medicine, Haraldsplass Deaconess Hospital, Bergen, Norway; ^7^UK Dementia Research Institute, Institute of Psychiatry, Psychology & Neuroscience, King's College London, London, United Kingdom; ^8^School of Cardiovascular Medicine and Sciences, British Heart Foundation Centre of Research Excellence, Faculty of Life Sciences and Medicine, King's College London, London, United Kingdom; ^9^Department of Food & Drug, University of Parma, Parma, Italy; ^10^Department of Cardiology, Stavanger University Hospital, Stavanger, Norway

**Keywords:** mild cognitive impairment, MCI, anthocyanins, lipids, inflammation markers

In the original article, there was a mistake in **Table 2** as published.

Due to a typographical error, the authors supplied incorrect *p*-values in **Table 2**, which resulted in subsequent errors in the results and discussion sections and the abstract. The main change is that there is no statistically significant between-group difference for ΔRANTES (difference from baseline to study end). Also, concerning ΔMCP-1 and Δfasting glucose statistically significant between-group differences emerged.

All of the incorrect data in **Table 2** have been revised and are written in ***bold*** ***italics***.

The corrected Table 2 appears below.

**Table 2:** Changes from baseline to 16 weeks follow-up in serum variables, for participants with supplementation (active) and for control participants.

	**Active (*n* = 27)**		**Control (*n* = 20)**		**Active vs. Control**
	**Median (IQR)**	p*	**Median (IQR)**	p*	***p^**#**^***
**Cholesterol (mmol/L)**			**Cholesterol**		
Pre	4.0 (3.1 to 5.5)		5.1 (4.5 to 5.5)		
Post	4.6 (3.3 to 6.0)		5.1 (4.6 to 5.6)		
Diff	0.2 (0.1 to 0.7)	0.009	0.1 (−0.2 to 0.5)	0.29	0.34
**HDL (mmol/L)**			**HDL**		
Pre	1.2 (1.0 to 1.4)		1.5 (1.1 to 1.7)		
Post	1.2 (1.1 to 1.5)		1.4 (1.2 to 1.8)		
Diff	0.0 (−0.1 to 0.1)	0.81	0.1 (−0.1 to 0.1)	0.21	0.23
**LDL (mmol/L)**			**LDL**^**n = 19**^		
Pre	2.4 (1.8 to 3.9)		3.3 (2.9 to 3.9)		
Post	3.0 (1.8 to 4.3)		3.3 (2.8 to 4.0)		
Diff	0.1 (−0.1 to 0.3)	0.21	0.0 (−0.1 to 0.4)	0.62	0.72
**Triglycerides (mmol/L)**			**Triglycerides**		
Pre	1.0 (0.7 to 1.4)		0.9 (0.6 to 1.3)		
Post	1.0 (0.7 to 1.7)		0.9 (0.6 to 1.8)		
Diff	0.1 (0.7 to 1.7)	0.016	0.0 (−0.1 to 0.4)	0.072	0.84
**Fasting glucose (mmol/L)**			**Fasting glucose**		
Pre	5.4 (4.9 to 5.6)		5.3 (5.0 to 5.6)		
Post	5.5 (5.3 to 6.3)		5.0 (4.8 to 5.7)		
Diff	0.2 (−0.1 to 0.4)	0.058	−0.2 (−0.4 to −0.03)	0.009	***0.003***
**HbA1c (%)**			**HbA1c**		
Pre	5.8 (5.6 to 6.1)		5.6 (5.4 to 5.8)		
Post	5.8 (5.6 to 6.1)		5.4 (5.2 to 5.6)		
Diff	0.0 (−0.1 to 0.1)	0.87	−0.05 (−0.2 to 0.0)	0.057	***0.26***
**IL-8 (mmol/L)**			**IL-8**		
Pre	9.0 (7.7 to 10.3)		7.5 (7.2 to 8.4)		
Post	9.2 (6.9 to 11.1)		7.8 (7.2 to 8.9)		
Diff	0.0 (−1.5 to 1.2)	0.80	0.2 (−1.0 to 1.5)	0.79	***0.71***
**MCP-1 (pg/mL)**			**MCP-1**		
Pre	42.2 (10.3 to 59.4)		51.7 (40.9 to 70.2)		
Post	41.3 (11.1 to 60.2)		52.8 (45.1 to 93.7)		
Diff	0.0 (−5.4 to 1.7)	0.55	1.9 (0.2 to 17.5)	0.014	***0.011***
**RANTES (pg/mL)**			**RANTES**		
Pre	9206 (8172 to 9833)		8800 (8370 to 9761)		
Post	8918 (8046 to 9942)		9164 (8651 to 10027)		
Diff	−161 (−730 to 677)	0.81	19.09 (−633 to 1105)	0.41	***0.41***
**TNFa (pg/mL)**			**TNFa**^**n = 19**^		
Pre	10.1 (7.8 to 13.3)		6.5 (6.1 to 10.9)		
Post	9.9 (6.5 to 13.9)		8.0 (5.8 to 11.4)		
Diff	0.9 (−2.8 to 2.9)	0.74	−0.4 (−1.5 to 3.9)	0.66	***0.95***

RANTES; CCL-5/RANTES (regulated on activation, normal T-cell expressed and secreted); Diff, median difference between baseline and follow up serum measurements; IQR, interquartile range; mmol/L, millimol/liter; pg/mL, picomol/liter. *The within group difference from baseline to study end. ^#^The between group differences for Δ (difference from baseline to study end).

Consequently, there was an error in the abstract:

“CCL-5/RANTES [regulated on activation, normal T-cell expressed and secreted (RANTES)]” should be “monocyte chemoattractant protein (MCP-1) and fasting glucose.”

A correction has been made to the abstract under subsection “Results”:

Results: There was a significant difference between groups for monocyte chemoattractant protein (MCP-1) and fasting glucose. In addition, total cholesterol and triglycerides were significantly increased in the AG. Improvements in memory and executive test scores were observed. No adverse effects were reported.

Also, there was an error in the result section:

“The only significant between-group difference was for ΔRANTES (difference from baseline to study end) which decreased in the supplementation group and increased in the NC group” should be “The only significant between-group difference was for difference were for ΔMCP-1 (difference from baseline to study end) (*p* = 0.011) and Δfasting glucose (*p* = 0.003).”

A correction has been made to the Section: Results, Paragraph 3:

The only significant between-group difference was for difference were for ΔMCP-1 (difference from baseline to study end) (*p* = 0.011) and Δfasting glucose (*p* = 0.003). (Table 2).

Finally, there were errors in the discussion section:

“There was a non-significant decrease in serum levels of RANTES in the AG and a non-significant increase in the NC during the study period. However, the between-group difference in Δ serum levels of RANTES was statistically significant.” should be “There was a non-significant increase in serum levels of MCP-1 in the AG and a significant increase in the NC during the study period. The between-group difference in Δ serum levels of MCP-1 was statistically significant.”

“In addition; There was a significant between-group difference for ΔRANTES, although anthocyanin supplementation did not significantly reduce RANTES in the AG. Still, our results are consistent with similar findings in a randomized, double-blind trial in hypercholesterolemic individuals consuming purified anthocyanins for 24 weeks (Song et al., 2014), and in a parallel-designed, placebo-controlled trial (Karlsen et al., 2007).” should be “As there was a significant between-group difference for ΔMCP-1, our results are partly consistent with findings in a randomized, double-blind trial in hypercholesterolemic individuals consuming purified anthocyanins for 24 weeks (Song et al., 2014), and in a parallel-designed, placebo-controlled trial (Karlsen et al., 2007).”

Corrections have been made to section: Discussion, Paragraphs 2 and 6:

Our findings are somewhat inconclusive. While some cognitive improvements were observed in the AG, there were no significant changes in serum levels of some risk factors for dementia; i.e., fasting glucose, HbA1c or pro-inflammatory cytokines. There was a non-significant increase in serum levels of MCP-1 in the AG and a significant increase in the NC during the study period. The between-group difference in1serumlevels of MCP-1 was statistically significant.

Regarding the inflammation markers, RANTES promotes activation and migration of leukocytes and mediates neuroinflammation and brain microvascular dysfunction (Appay and Rowland-Jones, 2001; Dénes et al., 2010; Yilmaz and Granger, 2010). As there was a significant between-group difference for 1MCP-1, our results are partly consistent with findings in a randomized, double-blind trial in hypercholesterolemic individuals consuming purified anthocyanins for 24 weeks (Song et al., 2014), and in a parallel-designed, placebo-controlled trial (Karlsen et al., 2007). Other studies did not report a reduction of pro-inflammatory mediators after anthocyanin supplementation (Hassellund et al., 2013; Kent et al., 2015). Therefore, the anti-inflammatory effect of anthocyanins and the potential to reduce neuroinflammation and brain microvascular dysfunction associated with cognitive decline in adults at risk of dementia (Grammas, 2011) should be studied in larger randomized studies.

The authors apologize for these errors and state that they do not change the scientific conclusions of the article in any way. The original article has been updated.

