# Supplementary material for: Effects of Anthocyanin Supplementation on Serum Lipids, Glucose, Markers of Inflammation and Cognition in Adults With Increased Risk of Dementia – A Pilot Study
Source: Front Genet. 2019 Jun 11;10:536. doi: 10.3389/fgene.2019.00536 (PMC6581024; doi:10.3389/fgene.2019.00536)
Supplement: FIGURE S1 — Changes from baseline to 16 weeks follow-up in serum lipids, for participants with anthocyanin supplementation. mmol/L, millimole/liter. [file Data_Sheet_1.pdf]

Supplementary Material

Figure S1

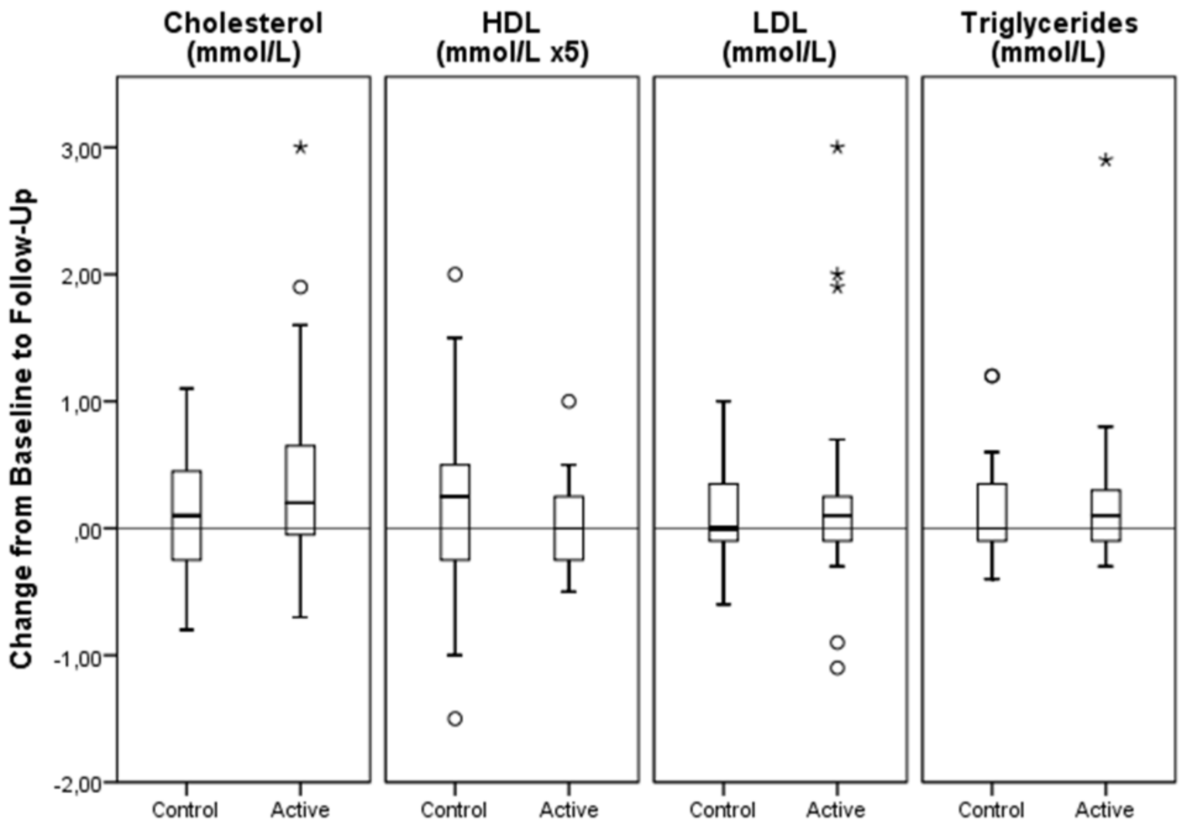

Figure S2

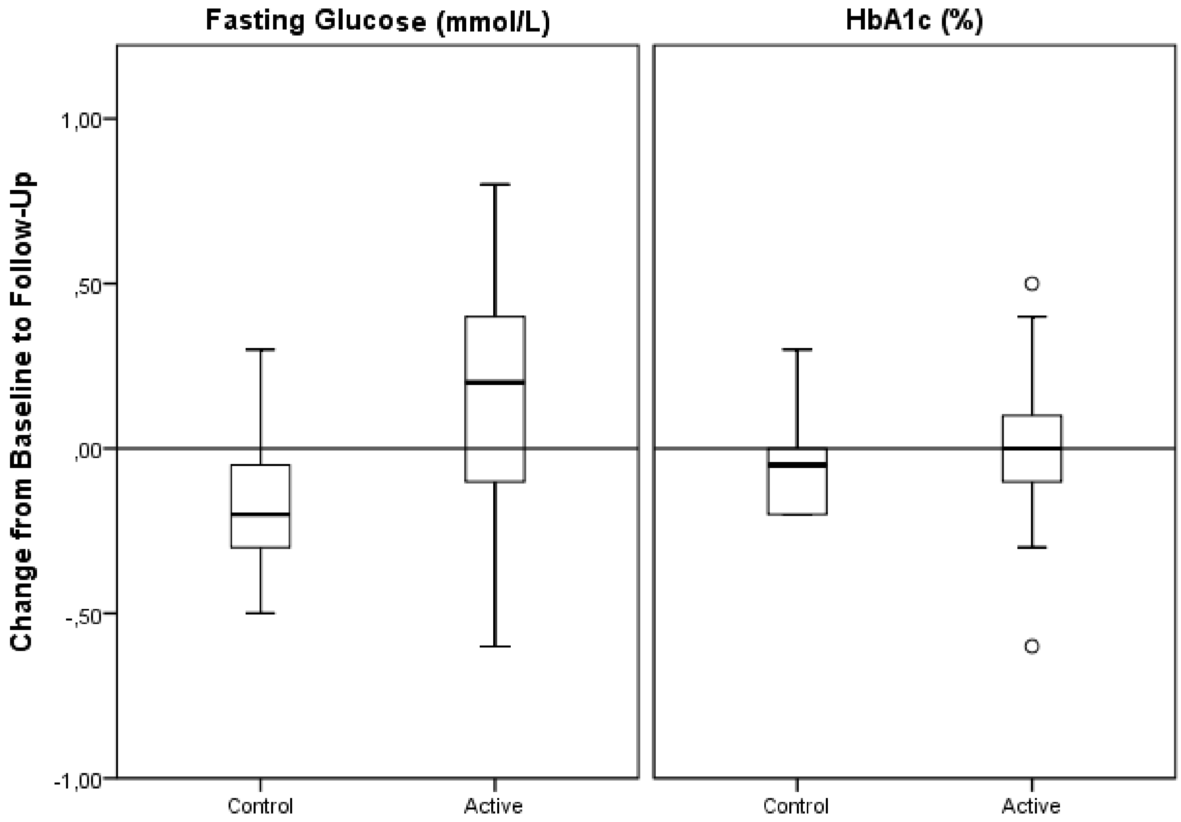
Fasting Glucose (mmol/L)
HbA1c (%)
Change from Baseline to Follow-Up
1,00
,50
,00
-,50
-1,00
Control
Active
Control
Active

Figure S3

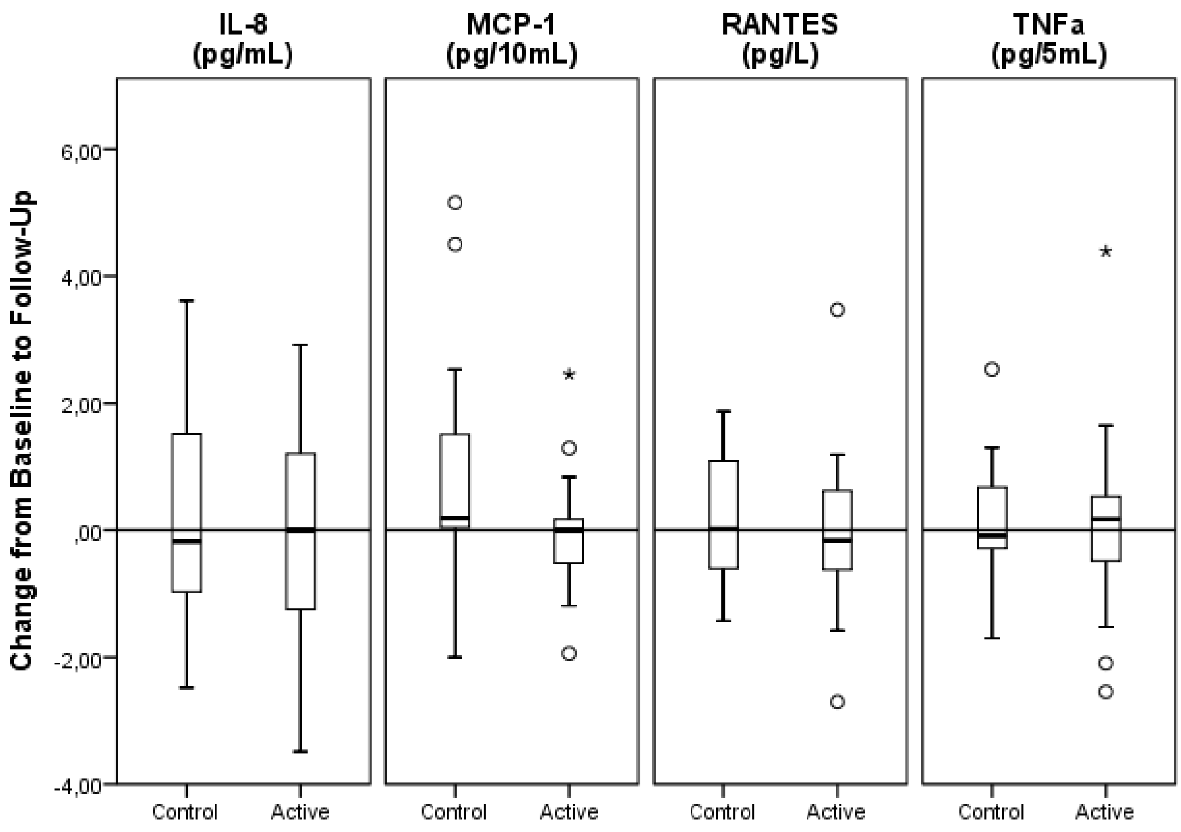
IL-8
(pg/mL)
MCP-1
(pg/10mL)
RANTES
(pg/L)
TNFa
(pg/5mL)
Change from Baseline to Follow-Up
6,00
4,00
2,00
,00
-2,00
-4,00
Control
Active
Control
Active
Control
Active
Control
Active

Figure S4

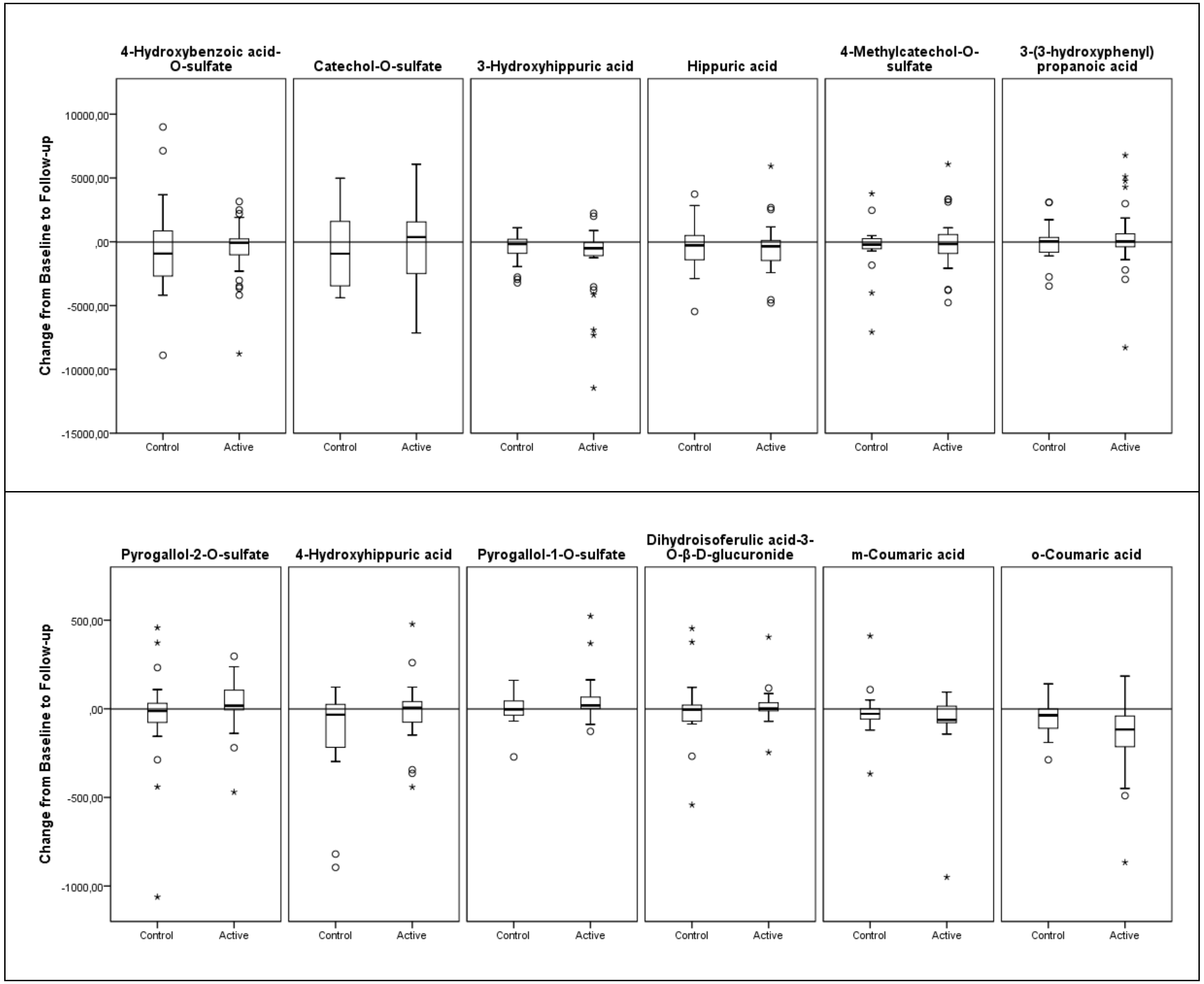
4-Hydroxybenzoic acid-O-sulfate
Catechol-O-sulfate
3-Hydroxyhippuric acid
Hippuric acid
4-Methylcatechol-O-sulfate
3-(3-hydroxyphenyl) propanoic acid
Change from Baseline to Follow-up
10000,00
5000,00
,00
-5000,00
-10000,00
-15000,00
Control
Active
Pyrogallol-2-O-sulfate
4-Hydroxyhippuric acid
Pyrogallol-1-O-sulfate
Dihydroisoferulic acid-3-O-β-D-glucuronide
m-Coumaric acid
o-Coumaric acid
500,00
,00
-500,00
-1000,00

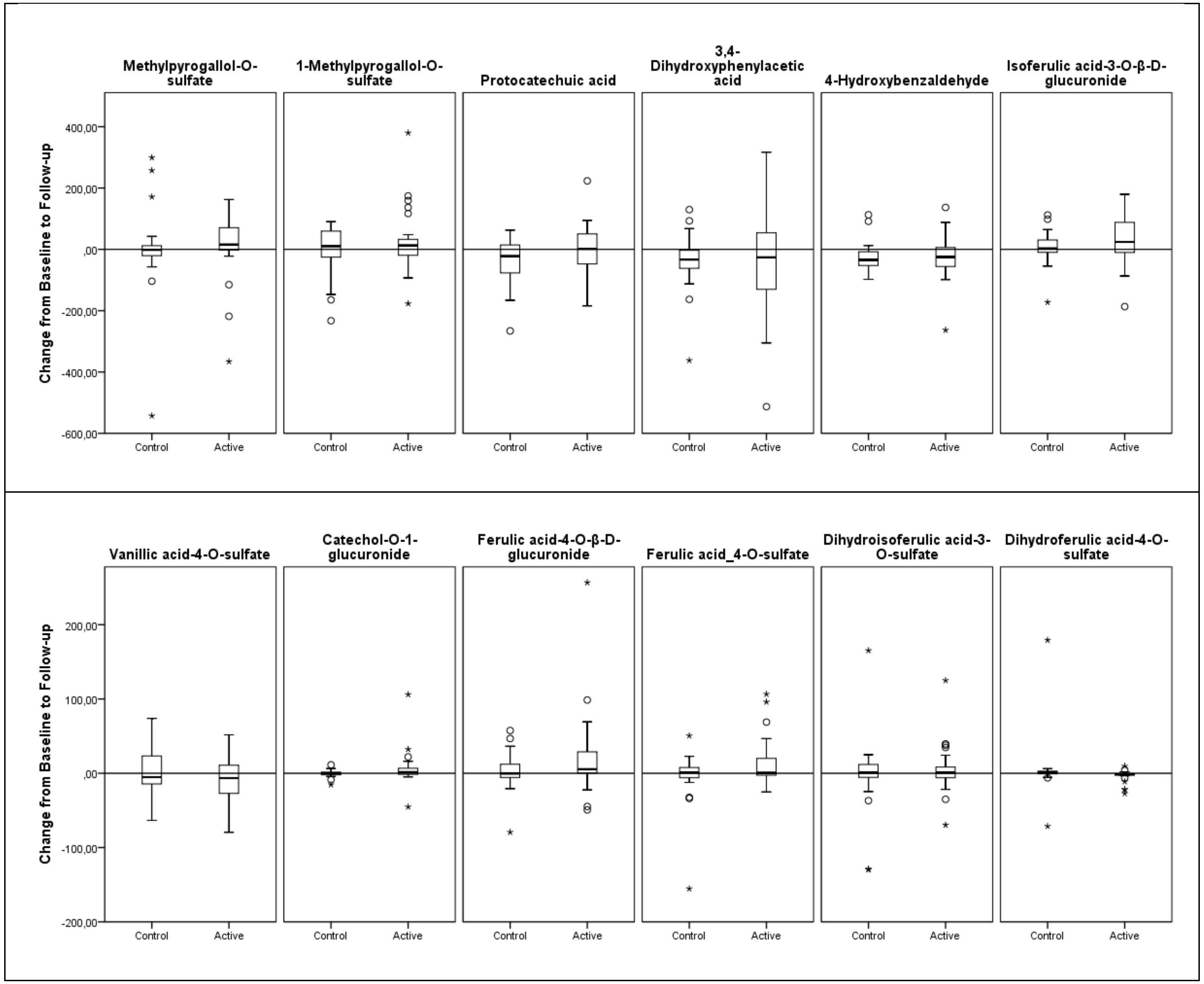
Methylpyrogallol-O-sulfate
1-Methylpyrogallol-O-sulfate
Protocatechuic acid
3,4-Dihydroxyphenylacetic acid
4-Hydroxybenzaldehyde
Isoferulic acid-3-O-β-D-glucuronide
Change from Baseline to Follow-up
400,00
200,00
,00
-200,00
-400,00
-600,00
Control
Active
Vanillic acid-4-O-sulfate
Catechol-O-1-glucuronide
Ferulic acid-4-O-β-D-glucuronide
Ferulic acid_4-O-sulfate
Dihydroisoferulic acid-3-O-sulfate
Dihydroferulic acid-4-O-sulfate
200,00
100,00
,00
-100,00
-200,00

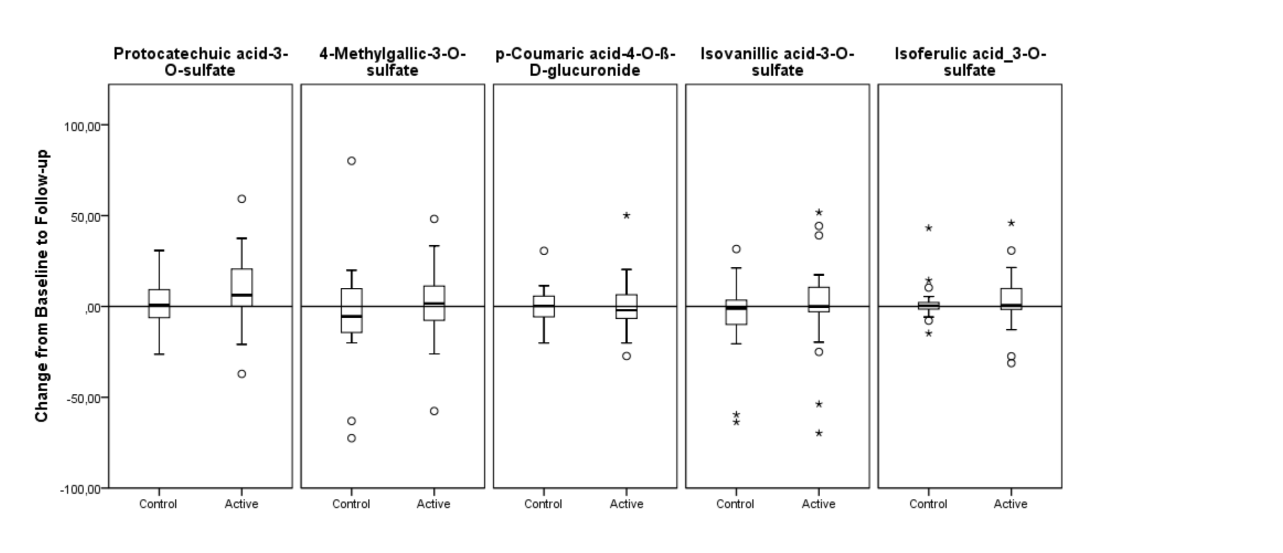
Protocatechuic acid-3-O-sulfate
4-Methylgallic-3-O-sulfate
p-Coumaric acid-4-O-ß-D-glucuronide
Isovanillic acid-3-O-sulfate
Isoferulic acid_3-O-sulfate
Change from Baseline to Follow-up
100,00
50,00
,00
-50,00
-100,00
Control
Active

Figure S5

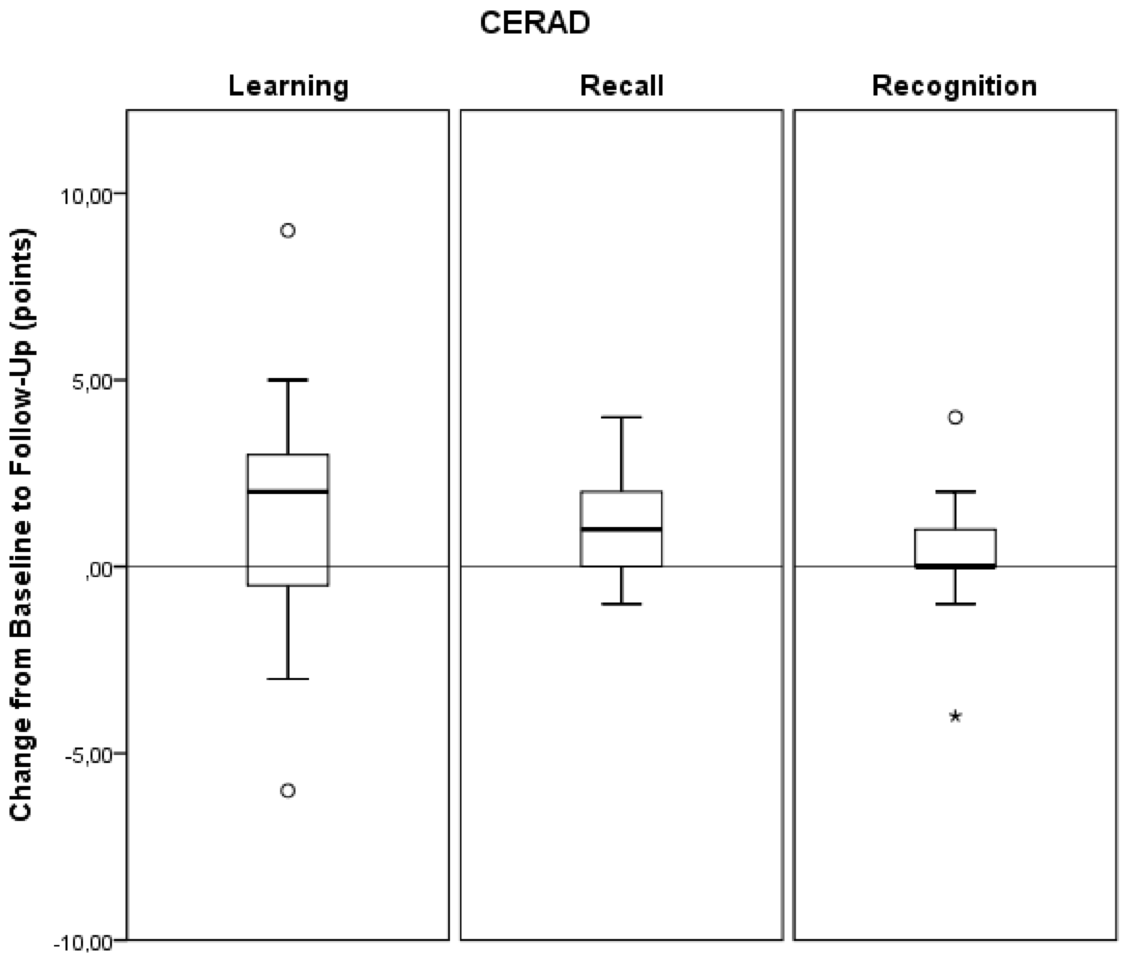
CERAD
Learning
Recall
Recognition
Change from Baseline to Follow-Up (points)
10,00
5,00
,00
-5,00
-10,00

Figure S6

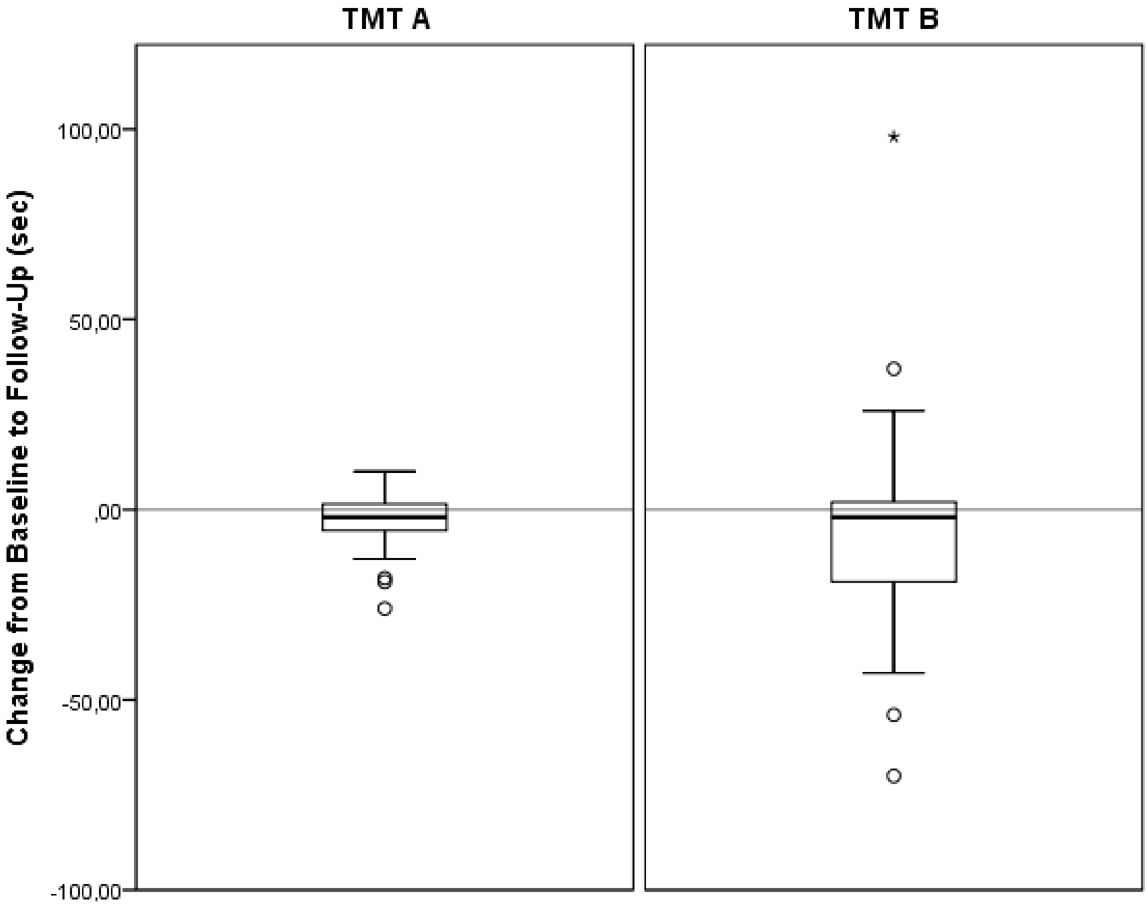
TMT A
TMT B
Change from Baseline to Follow-Up (sec)
100,00
50,00
,00
-50,00
-100,00

Figure S7

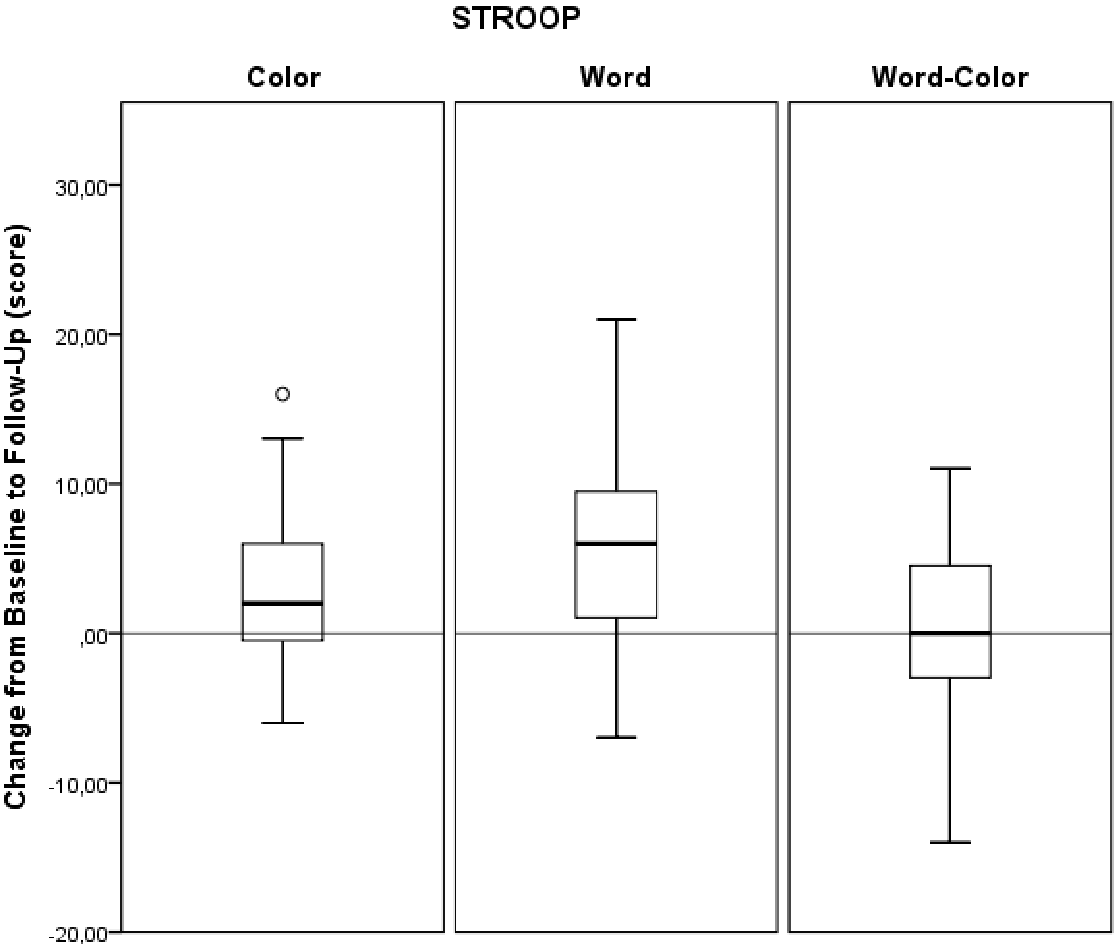
STROOP
Color
Word
Word-Color
Change from Baseline to Follow-Up (score)
30,00
20,00
10,00
,00
-10,00
-20,00

Table S1 Changes from baseline to 16 weeks follow-up in serum variables, for participants with supplementation (active) and for control participants.

	**Active (n = 27)**<br>$\bar{x}$ (SD, range)	P^*	**Control (n = 20)**<br>$\bar{x}$ (SD, range)	P^*	Active vs Control<br>$p^\$$
**Cholesterol**			**Cholesterol**		
Pre	4.3 (1.4, 1.3 to 6.7)		5.0 (0.8, 3.4 to 6.5)		
Post	4.7 (1.4, 2.6 to 7.3)		5.2 (0.8, 3.7 to 6.6)		
Diff	0.4 (0.8, -0.7 to 3.0)	0.01	0.1 (0.5, -0.8 to 1.1)	0.25	$0.14^\#$
**HDL**			**HDL**		
Pre	1.2 (0.3, 0.8 to 1.9)		1.5 (0.5, 1 to 2.8)		
Post	1.2 (0.3, 0.8 to 1.8)		1.5 (0.5, 0.9 to 2.8)		
Diff	0.0 (0.1, -0.1 to 0.2)	0.81	0.1 (0.2, -0.3 to 0.4)	0.20	$0.27^\#$
**LDL**			**LDL$^{n=19}$**		
Pre	2.8 (1.3, 1.2 to 5.1)		3.3 (0.9, 1.3 to 4.7)		
Post	3.1 (1.4, 0.9 to 5.4)		3.2 (0.8, 1.6 to 4.6)		
Diff	0.2 (0.8, -1.1 to 3)	0.16	0.1 (0.4, -0.6 to 1)	0.54	$0.37^\#$
**Triglycerides**			**Triglycerides**		
Pre	1.1 (0.5, 0.6 to 2.4)		0.8 (0.4, 0.4 to 2)		
Post	1.4 (0.5, 0.5 to 5.3)		1.2 (0.7, 0.4 to 3.2)		
Diff	0.2 (0.6, -3.5 to 2.9)	0.05	0.2 (0.4, -0.4 to 1.2)	0.067	0.74
**Fasting glucose**			**Fasting glucose**		
Pre	5.6 (0.8, 4.5 to 7.3)		5.3 (0.5, 4.6 to 6.6)		
Post	5.7 (0.7, 4.6 to 7.2)		5.2 (0.5, 4.3 to 6.3)		
Diff	0.1 (0.4, -0.6 to 0.8)	0.07	-0.2 (0.2, -0.5 to 0.3)	0.005	$0.002^\#$
**HbA1c**			**HbA1c**		
Pre	5.8 (0.5, 4.9 to 7.9)		5.5 (0.3, 4.8 to 6.2)		
Post	5.8 (0.5, 5.3 to 7.3)		5.4 (0.4, 4.6 to 6.2)		
Diff	-0.0 (0.2, -0.6 to 0.5)	0.88	-0.1 (0.1, -0.2 to 0.3)	0.076	0.33
**IL-8**			**IL-8**		
Pre	9.0 (2.1, 5.2 to 13.3)		7.9 (1.9, 5.54 to 13.86)		

Post	8.9 (2.5, 3.8 to 13.6)		8.1 (2.0, 4.7 to 12.9)		
Diff	-0.1 (1.7, -3.5 to 2.9)	0.75	0.1 (1.7, -2.5 to 3.6)	0.72	0.63
**MCP-1**			**MCP-1**		
Pre	42.7 (31.4, 5.8 to 152.5)		56.4 (24.4, 19.5 to 119)		
Post	42.1 (29.6, 6.0 to 133.1)		65.2 (31.2, 20.6 to 139)		
Diff	-0.7 (8.3, -19.4 to 24.6)	0.69	8.8 (16.8, -20 to 51.6)	0.030	0.028[#]
**RANTES**			**RANTES**		
Pre	9045 (1324, 5483 to 11846)		8971 (1098, 6855 to 11410)		
Post	9012 (1238, 6327 to 12559)		9159 (1297, 5527 to 11643)		
Diff	-33 (1118, -2705 to 3469)	0.88	189 (994, -1428 to 1867)	0.41	0.49
**TNFa**			**TNFa**$^{n=19}$		
Pre	11.1 (4.4, 3.3 to 24.1)		8.5 (3.6, 3.7 to 16.1)		
Post	11.4 (8.0, 4.2 to 46.1)		9.8 (4.7, 0.98 to 23)		
Diff	0.3 (6.5, -12.7 to 22)	0.85	0.7 (4.9, -8.54 to 12.7)	0.56	0.82

Table S2 Changes from baseline to 16 weeks follow-up in cognitive variables, for participants with supplementation (active).

	**Active (n = 27)**	
	$\bar{x}$ (SD, range)	P^*
**CERAD Learning**		
Pre	19.4 (4.6, 8 to 27)	
Post	20.8 (5.4, 8 to 29)	
Diff.	1.4 (3.0, -6 to 9)	0.021
**CERAD Recall**		
Pre	5.8 (2.2, 1 to 9)	
Post	6.9 (2.6, 1 to 10)	
Diff.	1.1 (1.8, -1 to 4)	< 0.001
**CERAD Recognition**		
Pre	18.7 (1.7, 14 to 20)	
Post	19.2 (1.6, 13 to 20)	
Diff.	0.5 (1.4, -4 to 4)	0.091
**TMT A**		
Pre	35.5 (12.5, 15 to 66)	
Post	32.3 (12.2, 13 to 64)	
Diff.	-3.2 (8.4, -26 to 10)	0.059
**TMT B**		
Pre	96.9 (47.2, 48 to 226)	
Post	91.0 (63.7, 47 to 321)	
Diff.	-5.9 (31.3, -70 to 98)	0.35
**STROOP Word**		
Pre	83.4 (17.16, 48 to 115)	
Post	88.8 (17.9, 49 to 119)	
Diff.	5.4 (6.1, -7 to 21)	< 0.001
**STROOP Color**		
Pre	58.9 (12.9, 26 to 83)	
Post	61.4 (12.9, 38 to 88)	
Diff.	2.5 (5.5, -6 to 16)	0.025
**STROOP Word-Color**		
Pre	34.0 (11, 11 to 56)	
Post	34.2 (12, 14 to 60)	
Diff.	0.2 (5.8, -14 to 11)	0.84
